# Utilization of a nanostructured lipid carrier encapsulating pitavastatin–*Pinus densiflora* oil for enhancing cytotoxicity against the gingival carcinoma HGF-1 cell line

**DOI:** 10.1080/10717544.2022.2155269

**Published:** 2022-12-12

**Authors:** Raed I. Felimban, Hossam H. Tayeb, Adeel G Chaudhary, Majed A. Felemban, Fuad H. Alnadwi, Sarah A. Ali, Jazia A. Alblowi, Eman ALfayez, Deena Bukhary, Mohammed Alissa, Safa H. Qahl

**Affiliations:** a Department of Medical Laboratory Sciences, Faculty of Applied Medical Sciences, King Abdulaziz University, Jeddah, Saudi Arabia; b Center of Innovation in Personalized Medicine (CIPM), 3D Bioprinting Unit, King Abdulaziz University, Jeddah, Saudi Arabia; c Center of Innovation in Personalized Medicine (CIPM), Nanomedicine Unit, King Abdulaziz University, Jeddah, Saudi Arabia; d Centre for Artificial Intelligence in Precision Medicines, King Abdulaziz University, Jeddah, Saudi Arabia; e Department of Nuclear Engineering, Faculty of Engineering, King Abdulaziz University, Jeddah, Saudi Arabia; f Department of Oral Diagnostic Sciences, Faculty of Dentistry, King Abdulaziz University, Jeddah, Saudi Arabia; g Department of Periodontology Faculty of Dentistry, King Abdulaziz University, Jeddah, Saudi Arabia; h Department of Oral Biology, Faculty of Dentistry, King Abdulaziz University, Jeddah, Saudi Arabia; i Department of Pharmaceutics, College of Pharmacy, Umm Al-Qura University, Makkah, Saudi Arabia; j Department of Medical Laboratory Sciences, College of Applied Medical Sciences, Prince Sattam bin Abdulaziz University, Al-Kharj, Saudi Arabia; k Department of Biology, College of Science, University of Jeddah, Jeddah, Saudi Arabia

**Keywords:** Pitavastatin, Pinus densiflora leaf, gingival cancer, experimental design, nanostructured lipid carrier

## Abstract

Oral squamous cell carcinoma (OSCC) is the most common epithelial tumor of the oral cavity. Gingival tumors, a unique type of OSCC, account for 10% of these malignant tumors. The antineoplastic properties of statins, including pitavastatin (PV), and the essential oil of the *Pinus densiflora* leaf (Pd oil) have been adequately reported. The goal of this investigation was to develop nanostructured lipid carriers (NLCs) containing PV combined with Pd oil and to determine their cytotoxicity against the cell line of human gingival fibroblasts (HGF-1). A central composite quadratic design was adopted to optimize the nanocarriers. The particle size and stability index of the nano-formulations were measured to evaluate various characteristics. TEM analysis, the entrapment efficiency, dissolution efficiency, and the cytotoxic efficiency of the optimized PV-loaded nanostructured lipid carrier drug delivery system (PV-Pd-NLCs) were evaluated. Then, the optimal PV-Pd-NLCs was incorporated into a Carbopol 940® gel base and tested for its rheological features and its properties of release and cell viability. The optimized NLCs had a particle size of 98 nm and a stability index of 89%. The gel containing optimum PV-Pd-NLCs had reasonable dissolution efficiency and acceptable rheological behavior and acquired the best cytotoxic activity against HGF-1 cell line among all the formulations developed for the study. The *in vitro* cell viability studies revealed a synergistic effect between PV and Pd oil in the treatment of gingival cancer. These findings illustrated that the gel containing PV-Pd-NLCs could be beneficial in the local treatment of gingival cancer.

## 1. Introduction

Oral malignancies, of which oral squamous cell carcinoma (OSCC) is the most common, are one of the most common tumors all over the world (Ferlay et al., 2015). Oral carcinoma is a major health worry, particularly in the developing world, in spite of the current techniques for cancer diagnosis and management (Rivera, 2015). OSCC is the most frequently encountered oral tumor and accounts for about 90% of oral cavity neoplasms (Casiglia & Woo, 2001). Interestingly, OSCC accounts for 4% of all cancers in males and 2% in females (Hoffman et al., 1998). Gingival neoplasms are an idiosyncratic subgroup of OSCCs, which account for approximately 10% of all OSCCs. They can simulate many benign oral lesions, particularly those of inflammatory origin, and this might result in delayed diagnoses and treatment. About 70% of gingival SCCs (GSCCs) occur in the mandible (Soo et al., 1988); they affect women more frequently than men (Barasch et al., 1994). GSCC has been clinically manifested, basically as an exophytic mass with a granulated or papillary surface or as an ulcerative lesion (Ramesh & Sadasivan, 2017). It is usually an asymptomatic, slow-growing lesion that is frequently misdiagnosed as a chronic inflammatory growth or a fibroma (Massano et al., 2006).

Statins are compounds from the drug class hydroxymethylglutaryl (HMG)-CoA reductase inhibitors. They prevent the synthesis of mevalonic acid from HMG-CoA, as implied by their name, thus upregulating hepatic low-density-lipoprotein (LDL) receptors, and can significantly decrease LDL cholesterol (Corsini et al., 1999). Based on this information, the efficiency of statins in managing cardiovascular diseases was well reported (Okazaki et al., 2004; Nakamura et al., 2006). Furthermore, some scientific reports showed that statins might potentially decrease the morbidity correlated with colorectal cancer (Yokomichi et al., 2017). Other research groups described the anticancer effects of statins, which might happen through the blocking of the HMG-CoA reductase pathway and cancer cell proliferation via adjusting the Rho, Ras, and Rac proteins (Di Bello et al., 2020). Statins were found to prohibit Ras prenylation, hence prompting the apoptosis of cancer cells via the depression of geranylgeranyl diphosphate (GGPP) biosynthesis (Tsubaki et al., 2017).

Pitavastatin (PV) is a statin with structural and pharmacokinetic properties that differ from other statins (Saito, 2011). PV has low metabolism via CYP enzymes and thus has minimum drug–drug interactions and offers prolonged safety and efficacy (Saito, 2009). Lately, a research group has demonstrated the anticancer mechanism of PV in *in vivo* therapeutic models employing OSCC cells. As claimed by the report, PV inhibits tumor growth by inhibiting AKT and ERK signals (Xu et al., 2021). Thus, investigating the antitumoral activity of HMG-CoA reductase inhibitors, including PV, is continually progressing, and promising new combination remedies are anticipated in the future.

The leaf of *Pinus densiflora*, which is a pine tree originally found in Asian mountains, has been extensively employed as a traditional medicine (Kim & Chung, 2000). The essential oil obtained from the plant’s leaves was reported to have good anti-inflammatory effects. They were attributed to its longifolene content, which was found to decrease levels of interleukin-4 (IL-4) and IL-13 and β-hexosaminidase during treatment of inflammatory-induced RBL-2H3 cells line of leukemia and their anti-inflammatory effect was found to be reasonable compared to that of dexamethasone, which is a potent anti-inflammatory steroidal drug (Yang et al., 2021). A group of investigators showed that *P. densiflora* essential oil had strong antiproliferative and proapoptotic actions on YD-8 cells and that such actions were mainly due to its reactive oxygen species (ROS)-dependent activation of caspases and Bcl-2 downregulation. It was strongly proposed that the essential oil extracted from the *P. densiflora* leaf had considerable anticancer activity against human OSCC (Jo et al., 2012).

Recently, nanotechnology applications were extensively investigated for either active or passive drug targeting by many administration routes (Elkomy et al., 2021; Alhakamy et al., 2022; Hussein et al., 2022; Salem et al., 2022). Nanostructured lipid carriers (NLCs) emerged as a new generation of lipid nanoparticles with fewer weaknesses than the first-generation nanoparticles, known as solid lipid nanoparticles (SLNs) (Xing et al., 2018; Chauhan et al., 2020). NLCs are manufactured using liquid and solid lipids that are characterized as biodegradable and compatible (López-García & Ganem-Rondero, 2015). The incorporation of oils in NLCs leads to structural defects in the solid lipids that result in a randomly arranged crystalline structure of the lipids; this precludes drug leakage and promotes drug entrapment in the carrier (Jain et al., 2017). Currently, NLCs have received a great deal of researchers’ attention as a good substitute for lipids, polymeric nanoparticles, nanoemulsions, and nanovesicles (Jaiswal et al., 2016). NLCs were exploited as an auspicious delivery system via different routes for various purposes, such as chemotherapy, gene therapy, brain targeting, and food preservation (Naseri et al., 2015). The advantages of NLCs include their abilities to entrap lipophilic and hydrophilic dugs, to control drug release, and to enhance drug stability and the fact that organic solvents are not necessarily needed in their formulations (Kaur et al., 2015).

A design of experiment (DOE) was used to obtain the most information from the fewest trials and, simultaneously, to clarify the effect of different variables and the interactions between them and elucidate why experimental errors might occur (Abou-Taleb et al., 2018). An interesting advantage of using such designs is that they require the strict and detailed following of statistical regulations, and this, in turn, forces investigators to be accurate in defining the aims and techniques needed to use them. Furthermore, the design can anticipate the characteristics of the optimum formulation and help with plans for how to fabricate it on large scale (Hosny KM et al., 2020). More importantly, the DOE is a profitable pathway of investigation since it offers the best solution for the formulation with minimum effort and expense (Khallaf et al., 2020).

Therefore, the current investigation aimed to incorporate PV plus the *P. densiflora* leaf essential oil in NLCs to obtain a prolonged drug release and test the formulation’s efficiency against the gingival carcinoma HGF-1 cell line.

## 2. Materials and methods

### 2.1. Materials

PV was obtained from Merck KGaA (Darmstadt, Germany). *P. densiflora* (Pd) essential oil was procured from the Tedia Company (Fairfield, OH, USA). Various other reagents and chemicals, such as glyceryl distearate (Precirol®), caprylocaproyl polyoxyl-8 glyceride (Labrasol®), propylene glycol monocaprylate (Capryol 90®), and a mixture of glyceryl ricinoleate and ethoxylated fatty alcohols (Ovucire®), were obtained from Gattefosse (Saint-Priest, France). Ethanol, chloroform, acetonitrile, and methanol were purchased from Sigma-Aldrich (St. Louis, MO, USA). The HGF-1 cell lines were a gift from the Ajia Biological Laboratories (Cairo, Egypt). All other solvents, chemicals, and reagents used were of analytical grade.

### 2.2. Experimental design for the selection of an optimized PV-Pd-NLCs

In the current study, the NLCs were optimized using a central composite quadratic design (Design-Expert software v. 13.0.5.0; Stat-Ease, Inc., Minneapolis, MN, USA). The design was used to select the optimal NLC formulation with the best levels of three factors: the lipid:PV ratio (*A*), Pd:Ovucire® ratio (*B*), and Labrasol® concentration (*C*). The particle size (*Y*_1_) and stability index (*Y*_2_) were chosen as the measurable responses (Table 1).

**Table 1.** The uncoded and coded levels of the NLCs formulation factors along with their constraints.

Independent variables	Level		
	–1	0	+1
*A*: Lipid:PV ratio	5:1	10:1	15:1
*B*: Pd:Ovucire ratio	1:9	2.5:7.5	4:6
*C*: Labrasol concentration (%)	1	2	3
Dependent variables	Constraints		
*Y*_1_: Particle size	Minimum		
*Y*_2_: Stability Index	Maximum		

### 2.3. Testing of PV solubility in different lipids

Each of the five lipids tested (i.e. Ovucire®, Precirol®, Compritol 888 ATO®, Geleol®, and Suppocire) was added to duplicate glass vials mixed with excess amount of PV, and the vials were vigorously vortexed before being incubated at 40 °C for the duration of the solubility research. To keep the medicine suspended, vials were regularly vortexed and carefully sealed. Samples were taken at 24, 48, and 72 h to attain equilibrium. The vials were whirled in a temperature-controlled centrifuge for 30 min at 5000 rpm, before sampling (Eppendorf centrifuge 5804R). The supernatant was put into a tared 5-mL volumetric flask, and diluted with 66% vol/vol chloroform and methanol mixture (Persson et al., 2013).

On a Shimadzu LC-10ATVP pump, SPD-M10 AVP with PDA detector, HPLC analysis of PV in collected samples was carried out. At room temperature, separation was accomplished using the phenomenex C18 (250 × 4.60); 5 particle size column and a mobile phase made up of 0.5% acetic acid and acetonitrile (35:65) at a flow rate of 1 mL/min. Prior to use, the mobile phase was filtered through a 0.45-m nylon filter (Millipore, USA) and sonicated to remove any gas. At 266 nm, the detection was carried out. A 20-loop sample injector valve was used to introduce the standard and sample. CLASS-VP software was used to measure peak areas during the data collecting process (Kumar et al., 2019).

### 2.4. Preparation of PV-Pd-NLCs

A previously published hot homogenization and sonication method was adopted for the development of the different PV-Pd-NLCs formulations (Chauhan et al., 2020). In brief, predetermined quantities of Ovucire®, Pd oil, Capryol 90®, and PV (10 mg), which comprised the lipid phase, were dissolved in a 1:1 mixture of chloroform and methanol. A rotary evaporator (R 10 BÜCHI Labortechnik AG, Flawil, Switzerland) was used to eliminate the organic solvent mixture. Then, the drug’s lipid layer was melted at 65–70 °C. In the meantime, predefined amounts of Labrasol® were dissolved in distilled water and then this aqueous phase was warmed at 65–70 °C. The warmed aqueous phase was decanted into the lipid phase, and the mixture was homogenized (IKA Homogenizer T18; IKA, Karnataka, India) at 6,000 to 24,000 rpm for 2 min. The developed emulsion was sonicated using a probe sonicator (Sonics Vibra-Cell VCX 750; Sonics and Materials, Inc., Newtown, CT, USA) for 1 min, and the NLCs that resulted were kept at 25 °C. The prepared PV-Pd-NLCs were lyophilized (Martin Christ GmbH, Osterode am Harz, Germany) at a condenser temperature of –45 °C and pressure of 7 × 10^−2^ mbar using mannitol as a cryoprotectant for 24 h and kept for further evaluation.

### 2.5. Characterization of prepared PV-Pd-NLCs

#### 2.5.1. Particle size determination

The dynamic light scattering method was applied to measure the particle size of the nanoformulations using a Zetatrac particle size analyzer (Microtrac, Inc., York, PA, USA). One milliliter of each lyophilized sample – which was first reconstituted with 5 mL double distilled water – was diluted with 9 mL of distilled water and agitated for 3 min. Then, 1 mL of each diluted sample was used to determine the particle size and polydispersity index (PDI) of each NLCs formulation (Hosny KM et al., 2021).

#### 2.5.2. Stability index determination

First, the reconstituted formulations were subjected to cool–warm stability testing, in which they were kept for 48 h at 4 °C and for another 48 h at 40 °C. This cycle was repeated three times, and the tested formulations were examined visually for signs of instability. The formulations that passed the cool–warm cycle examination were subjected to varying temperatures to affirm their thermodynamic stability (Rizg et al., 2022). The PV-Pd-NLCs went through three consecutive freeze–thaw cycles (i.e. freezing at –25 °C for approximately 12 h and thawing at 25 °C for another 12 h). Next, the samples were tested for droplet size. The samples’ stability index was estimated by comparing the collected particle size data with that obtained in initial measurements using the following equation (Hosny K et al. 2021):

Stability index = ((Initial size – Change in size)/Initial size) × 100 (1)

### 2.6. Selection of optimized PV-Pd-NLCs

The central composite quadratic design was used to optimize the NLCs, and it was done according to the constraints and aims listed in Table 1. Following the analysis of the findings for the particle size and stability index, the optimal NLCs was selected. The optimal PV-Pd-NLCs was tested for various parameters, such as ultramicroscopic characteristics, *in vitro* dissolution efficiency, and in vivo efficacy.

### 2.7. Ultramorphological analysis of optimal PV-Pd-NLCs

Transmission electron microscopy (TEM) was applied to detect the morphological and structural features of the optimal PV-Pd-NLCs (TEM H7500; Hitachi, Japan). The sample was first diluted up to 200-fold using double distilled water. Then, a drop of the diluted sample was placed on a copper grid supported by Formvar films. Any excess of the sample was removed by filter paper. Next, the sample was stained with 0.5% phosphotungstic acid solution for 30 s and set aside to dry. The instrument was operated at 80 kV for point-to-point resolution (Salem et al., 2020).

### 2.8. Determination of entrapment efficiency of optimal PV-Pd-NLCs

To determine the entrapment efficiency (EE%), samples of the optimal PV-Pd-NLCs (4 mL in volume) were centrifuged (3K30 Centrifuge, Sigma Centrifuges, SciQuip Ltd., Newtown, Wem, Shropshire, UK) at 20,000 rpm for 60 min. The unentrapped PV was estimated spectrophotometrically in the clear supernatant at 266 nm. The amount of entrapped PV was determined by subtracting the amount of free drug from the total amount of drug initially included in the formulation (Salem et al., 2020).

(2) $$\text{EE}\%\;=\frac{\text{Total}\;\text{drug}\;\text{amount}-\text{free}\;\text{drug}}{\text{total}\;\text{drug}\;\text{amount}}\times 100$$

### 2.9. Determination of *in vitro* release

An automated vertical Franz diffusion cell (Hanson Research Microette Plus, Chatsworth, CA, USA) was used for the estimation of the *in vitro* dissolution behavior of the optimal formulation in comparison to the drug suspension. A dialysis tubing cellulose membrane with a pore size of 14,000 Da and diffusion area of 1.7 cm^2^ was the barrier membrane mounted between the donor and receptor compartments. This membrane was first soaked in phosphate buffered saline (PBS, pH 6.8) at 25 + 0.05 °C for 15 min. Furthermore, 25 mL of PBS containing 0.1% of Tween 80 (i.e. dissolution medium) was inserted in the cell’s receptor compartment and stirred constantly using a magnetic stirrer to guarantee the sink condition. In the meantime, known weights of lyophilized NLCs were inserted in the donor compartment. At certain predefined intervals (0.5, 1.00, 2.00, 4.00, 6.00, 8.00, 10.00, and 12.00 h), samples of 2 mL were collected and the PV concentration was determined spectrophotometrically (UV/Visible Spectrophotometer, Jenway 6705, Vernon Hills, IL, USA) at a maximum wavelength of 266 nm. The dissolution efficiency was calculated as a percentage by comparing the quantity of PV in the optimal NLCs. This was determined before performing the dissolution analysis via an indirect method (Hosny KM et al., 2022). Briefly, samples of 4 mL of the optimal PV-Pd-NLCs were centrifuged (3K30 centrifuge, Sigma Centrifuges, SciQuip Ltd.) at 20,000 rpm for 60 min. The unentrapped PV was analyzed spectrophotometrically in the clear supernatant at 266 nm. In addition, the amount of entrapped drug was calculated by dividing the actual amount of drug loaded by the amount of theoretical drug loaded. These studies were carried out in triplicate for every formulation.

### 2.10. Preparation and characterization of the optimized PV-Pd-NLCs gel

To prepare a PV-Pd-NLCs gel, a certain amount of the optimal PV-Pd-NLCs that contained 100 mg/g of PV was dispersed in 10 mL of double distilled water. Next, 1% Carbopol 940 and 0.1% propylparaben were sprinkled in the prepared dispersions and stirred at 200 rpm until the Carbopol 940 was completely dispersed (Rizg et al., 2022). The dispersion was treated with a certain amount of triethanolamine to obtain a pH of 5.5 to ensure homogenous distribution and dissolution of the polymer. Entrapped air bubbles were removed by storing the PV-Pd-NLCs nanogel for 24 h in a refrigerator and keeping it for further characterization. The PV content of the nanogels was estimated by dispersing 1 g of the gels in 9 mL of methanol. The dispersion was sonicated in a bath sonicator for 10 min. The PV content in the produced dispersion was evaluated by measuring the UV absorbance at 266 nm. Various PV-loaded nanogels (shown in Table 2) were developed in the same way (Dantas et al., 2016).

**Table 2.** Details of samples prepared for the characterization and evaluation of the PV-Pd nanogels.

Formulation	Composition
A	Hydrogel containing optimized PV-Pd-NLCs
B	Hydrogel containing NLCs formulated with castor oil instead of Pd oil
C	Hydrogel containing with Pd-NLCs formulated without PV
D	Aqueous dispersion PV-Pd-NLCs formulated without Carbopol 940 gelling agent
E	Physical mixture of PV and Pd oil
F	Carbopol 940 hydrogel (plain)

#### 2.10.1. Rheological evaluation of the optimized PV-Pd-NLCs nanogel

The rheological characteristics of the optimal PV-Pd-NLCs nanogel (formulation A) were compared with those of the plain Carbopol hydrogel (formulation F). A Brookfield viscometer’s spindle 52 was the instrument employed in the experiment at 25 ± 1 °C. One gram of each sample was subjected to shear rates of 2, 10, 20, 30, 40, 50, and 60 s^−1^. The flow curves of the investigated nanogels were assembled, and Farrow’s constant (*n*) for each formulation was estimated using the following equation (El-Leithy et al., 2010):

 Log  G=n  Log  F– Log  η (3)where *G* is the shear rate, *η* is the viscosity, *F* is the shear stress, and *n* is the Farrow’s constant.

#### 2.10.2. *In vitro* release behavior of PV from different gel formulations

An *in vitro* drug release study was performed for different formulations, including (1) a hydrogel containing optimal PV-Pd-NLCs (A); (2) an aqueous dispersion of the optimal PV-Pd-NLCs without the Carbopol 940® gelling agent (D); and (3) an aqueous dispersion of 1% PV. In this experiment, a USP dissolution apparatus (type I, basket type; DT 700 LH Device, Erweka GmbH DT 700, Heusenstamm, Germany) was utilized. First, the studied formulations were inserted in separate glass cylindrical tubes with a diameter of 2.7 cm and a length of 6 cm. The release media which was isotonic phosphate buffer (250 ml, pH 6.6) was blended at 50 rpm and maintained at 37 ± 0.5 °C. The experiment continued for 180 min (i.e. 3 h) and samples were withdrawn at certain time intervals (5, 30, 60, 90, 120, 150, and 180 min). Next, the PV release percentages at certain time intervals were assigned by measuring the absorbance at 266 nm. All measurements were performed in triplicate, and the data were presented as means ± standard deviations (SD).

### 2.11. *In v itro* cell viability assay

As per a formerly depicted method, the 3-(4,5-dimethylthiazol-2-yl)-5-(3-carboxymethoxyphenyl)-2-(4-sulfophenyl)-2*H*-tetrazolium dye, aka, MTS was used to estimate the *in vitro* cytotoxic behavior of the prepared formulations against the human gingival fibroblast HGF-1 cell line (Tsugeno et al., 2014). As outlined in Table 2, the hydrogel that contained the optimized PV-Pd-NLCs (formulation A) was compared with formulations B, C, D, E, and F. The freshly explanted tumor tissues were employed as a subculturing medium for the migrating and proliferating epithelial cells. Incubators containing T-25 culture vessels (Nunc) were utilized for the breeding of gingival fibroblasts HGF-1 (CRL-2014, ATCC) at 37 °C in an atmosphere of 5% CO_2_. DMEM (ATCC) enriched with 10% FBS (Sigma-Aldrich) was the employed growth medium. Following incubation for 24 h of the gingival fibroblast cultures, the cells were rinsed three times. The tests were performed in three runs using culture Lab-Tek Chamber Slides (Nunc); they used either growth medium alone (i.e. control: 0.5 × 10^6^ HGF-1 cells) or other examined formulations (in concentrations of 0.1, 0.5, 1, 1.5, 2.5, 3.5, or 5 mM/L/0.5 × 10^6^ HGF-1 cells). The samples were incubated for 20 h at 37 °C in the presence of 5% CO_2_. In addition, the samples were incubated with 1 mM/L of the tested formulations for 5 h and 10 h. After the incubation period was over, the cells were washed with the culture medium and their viability was assessed. To perform this test, a fluorescence microscope (Nikon Eclipse E200, magnification of ×1000) was used, and the absorbance was evaluated with a microplate reader (Biotek Synergy, Santa Clara, CA, USA) at 492 nm. Finally, the dose–response curve was constructed, and from it the half-maximal inhibitory (IC_50_) concentrations were assessed. The antiproliferative activity of the best-performing formulation was tested against normal HGF-1 cells.

### 2.12. Lactate dehydrogenase enzyme release measurement

Lactate dehydrogenase (LDH) release test was performed using CytoTox 96 Non-Radioactive Cytotoxicity Assay kits (Promega Corp., Madison, WI, USA) (Szkaradkiewicz et al., 2014). HGF-1 gingival fibroblast cultures that were incubated for 24 h were used after being rinsed three times. The test was conducted using growth medium alone (i.e. control: 105 HGF-1 cells) and other tested formulations with concentrations of 0.1, 0.5, 1, 1.5, 2.5, 3.5, or 5 mM/L/10^5^ HGF-1 cells. The prepared cells were incubated for 20 h at 37 °C in the presence of 5% CO_2_, and the tubes were centrifuged at 500 rpm for 4 min at 20 °C. The tests were performed as per the manufacturer’s instructions. The results were recorded as the absorbance at 492 nm. The cytotoxicity percentage was calculated by dividing the absorbance value for the LDH release in the experimental samples by the absorbance values for the LDH release in the samples with maximum lysis.

## 3. Results and discussion

### 3.1. Determination of PV solubility in different lipids

**Figure 1.**
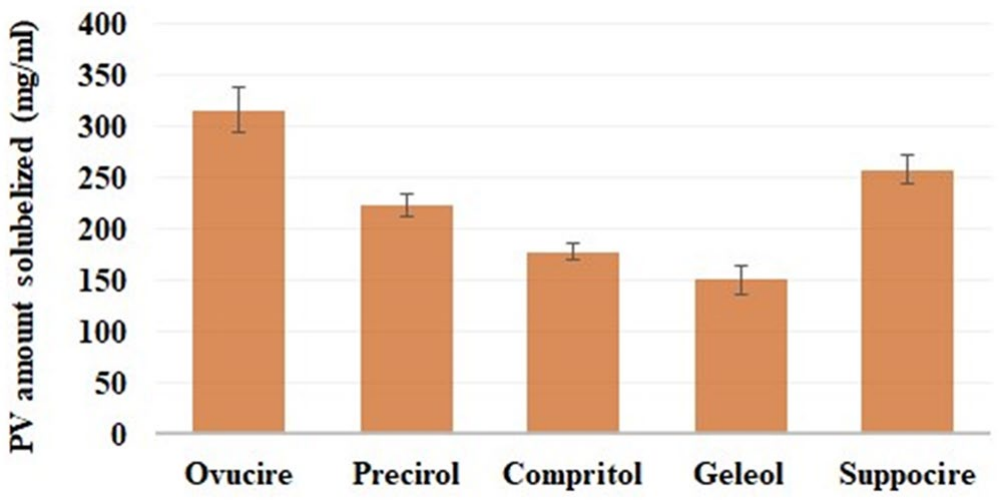
PV solubility in different lipids (mean ± SD).

The PV solubility was examined in the lipids Precirol®, Compritol®, Suppocire®, Geleol®, and Ovucire® (Figure 1). The PV exhibited the highest solubility in Ovucire (315 ± 10 mg). Therefore, Ovucire® was the lipid of choice for the lipid phase of the proposed NLCs.

### 3.2. Evaluation of the PV-Pd-NLCs

#### 3.2.1. Determination of the particle size of the PV-Pd-NLCs

The particle size of the nanocarriers fluctuated between 72 ± 1.5 and 120 ± 5.1 nm, as shown in Table 3, with PDI values from 0.1 to 0.29. Such outcomes suggest the adequate stability and uniformity of size distribution of the nanocarriers which will decrease the chance of particles precipitation.

**Table 3.** Central composite quadratic design and responses of PV-Pd-NLCs.

Run	*A*: Lipid:PV ratio	*B*: Pd:Ovucire® ratio	*C*: Labrasol® concentration	Particle size (*Y*_1_, nm)	Stability Index (*Y*_2_, %)	PDI
1	–1.681	0	0	78 ± 2.1	81 ± 1.0	0.12
2	1	1	–1	116 ± 3.0	79 ± 1.5	0.18
3	0	0	1.681	72 ± 1.5	75 ± 0.5	0.21
4	0	–1.681	0	101 ± 2.7	91 ± 2.0	0.19
5	1	1	1	99 ± 1.8	73 ± 1.5	0.22
6	1	–1	–1	127 ± 2.0	93 ± 3.0	0.26
7	–1	–1	1	86 ± 3.5	78 ± 1.3	0.18
8	0	0	0	98 ± 2.2	80 ± 1.5	0.17
9	0	0	0	99 ± 4.3	80 ± 2.0	0.15
10	1	–1	1	105 ± 1.9	79 ± 1.1	0.25
11	0	0	–1.681	120 ± 5.1	87 ± 2.0	0.29
12	0	0	0	97 ± 4.5	80 ± 0.5	0.11
13	–1	1	–1	103 ± 3.7	77 ± 2.1	0.16
14	–1	–1	–1	102 ± 3.5	90 ± 1.9	0.28
15	–1	1	1	84 ± 2.8	73 ± 1.7	0.26
16	0	0	0	98 ± 2.2	83 ± 1.9	0.17
17	1.681	0	0	120 ± 5.0	81 ± 1.1	0.22
18	0	0	0	99 ± 3.3	80 ± 0.8	0.23
19	0	0	0	97 ± 4.0	82 ± 0.4	0.18
20	0	1.681	0	100 ± 5.5	74 ± 0.5	0.29

A linear model of polynomial analysis determined the best significant squared mean value, and it surpassed the residual error (*p* < .0001); hence, it was endorsed for the analysis of the particle size data. The selected experimental design was investigated to show the efficiency of the effect of the lipid:PV ratio (*A*), Pd: Ovucire® ratio (*B*), and Labrasol concentration (*C*) on the PV-Pd-NLCs’ particle size. The model achieved an adjusted *R*^2^ value of 0.8997, which was in harmony with a predicted *R*^2^ value of 0.8499. An ANOVA analysis of the data used the following formula:

(4) Particle size= +100.05+10.44A−1.44B−11.33C

As can be noted from the above formula, factor *A*, which is the lipid:PV ratio, had a significant positive effect on the particle size (*p* value <.0001), while factor (*C*), which is the ratio of the surfactant Labrasol, had a considerable negative effect on the particle size. It was noted that factor (*B*), the Pd:Ovucire® ratio, exerted no effect on the nanocarrier’s particle size (*p* value = .2379).

The observed increase in particle size with the increase in the lipid:drug ratio could be based on the fact that a nanoparticle’s size is basically determined by its lipid content and that the lipid is likely to become agglomerated at high levels. This phenomenon can be understood in light of Stokes’ law, which states that such behavior might be due to the variation in density between the internal and external phases (Leroux et al., 1995). A group of investigators found that an increase in the amount of solid lipid resulted in the formation of flakes (Patel et al., 2005). Another research group reported that the particle size was liable to increase after the lipid/surfactant mass ratio increased and that the decrease of this ratio resulted in smaller particle sizes (Sarmento et al., 2006). In another investigation, scientists found that the higher the lipid concentration, the larger the particle size (Arora et al., 2011). In addition, an increase in the lipid:PV ratio might raise the viscosity of the formulation. If so, the speed of homogenization used during nanocarrier development would be lowered and the particle size would therefore be larger (Hu et al., 2005).

**Figure 2.**
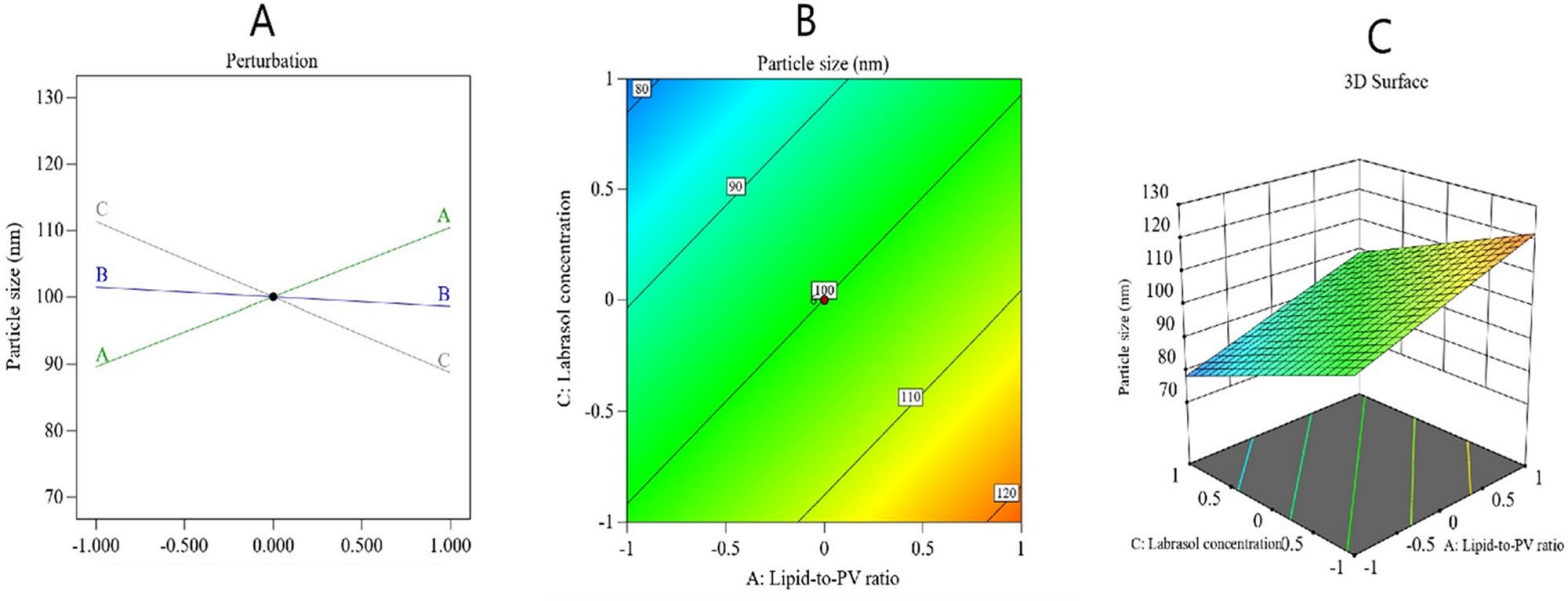
Perturbation diagram (A), contour plot (B), and 3D response surface plot (C) showing the effect of the studied factors on the particle size.

An increase in the Labrasol® ratio decreased the particle size of NLCs. Such a decrease could be due to the ability of Labrasol® to lower the interfacial tension between the organic and inorganic phases, leading to increased fluidity of NLCs membranes and, hence, a decrease in the particle size of the NLCs (Muller, 2007; Nazar et al., 2010). Figure 2 presents the main effect diagram and the contour and three-dimensional surface plots, which show the effect of the studied factors on the PV-Pd-NLCs particle size.

#### 3.2.2. Stability index determination

The stability index is a substantial aspect of the overall stability of the NLCs. The stability index (*Y*_2_) of the nanoformulations was calculated as 73 ± 1.7 to 91 ± 2.0%, as summarized in Table 3. By analyzing the collected data, it was found that *Y*_2_ followed a 2FI model of polynomial analysis. The model had an adjusted *R*^2^ value of 0.9471, which was very close to the expected *R*^2^ value of 0.8998. The analysis of variance of the collected data yielded formula (5)

(5) Stability index= +80.80+0.4393A−4.88B−4.11C+0.2500AB−0.5000AC+2.00BC 

**Figure 3.**
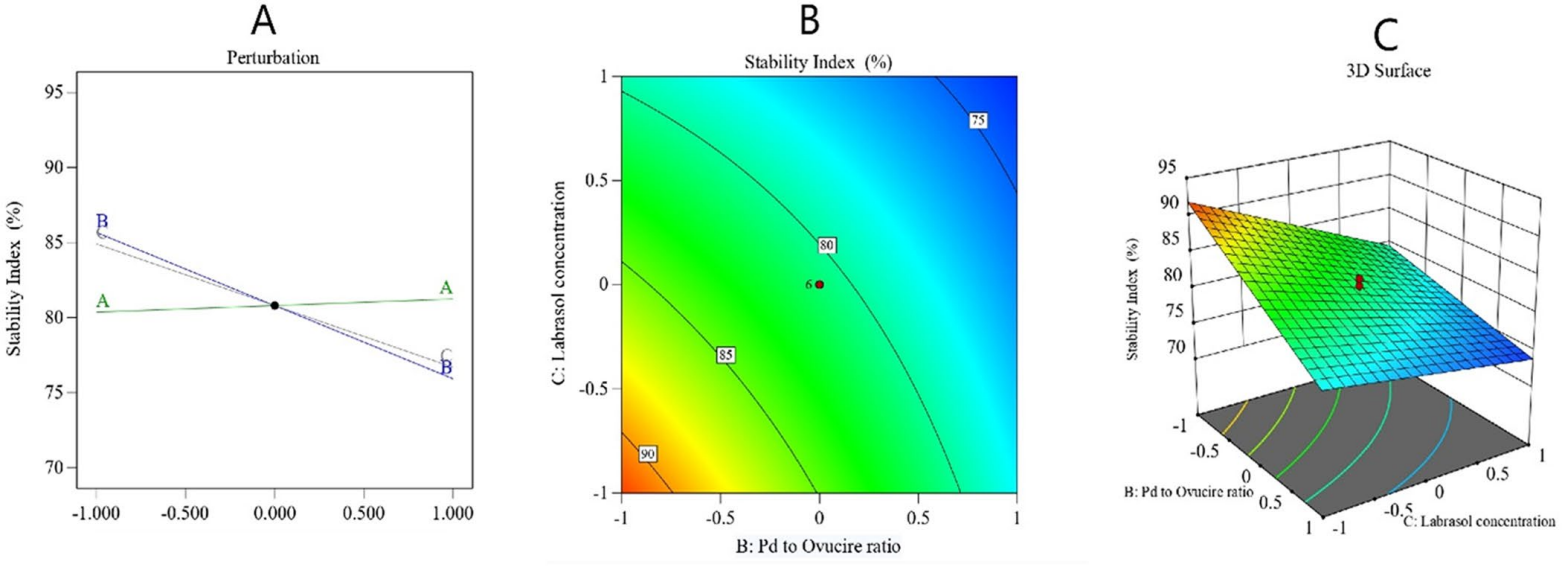
Perturbation diagram (A), contour plot (B), and 3D response surface plot (C) showing the effect of the studied factors on the stability index.

As per the obtained formula, factor *A* had a nonsignificant effect on the stability index, while factors *B* and *C* exerted a significantly negative effect on the same parameter (*p* < .0001). The decrease in stability with the increase in factor *B* could be due to the expected decrease in the rigidity of the produced NLCs because the amount of Ovucire® would have been lowered; this lipid keeps particles hard and decreases the chance of particles to fuse and become agglomerated; hence, it preserves particles’ stability (Azhar Shekoufeh Bahari & Hamishehkar, 2016) and thus its decrease would result in a low stability. The unexpected decrease in the stability index with the increase in the Labrasol® ratio might be explained by the Ostwald ripening phenomenon, in which there is the growth of larger particles at the expense of smaller ones due to the mass transfer of the dispersed phase into the surrounding dispersion medium (Ee et al., 2008). Some investigators have reported that the Ostwald ripening mechanism increases with the increase of surfactant concentration because the particle size becomes smaller following such an increase and, hence, more particles pass into the surrounding medium, contribute to the formation of larger particles, and eventually decrease the stability (Witayaudom & Klinkesorn, 2017). Figure 3 presents the main effect diagram and the contour and 3-D surface plots, which show the effect of the studied factors on the PV-Pd-NLCs stability index.

### 3.3. Optimization of the PV-Pd-NLCs

**Figure 4.**
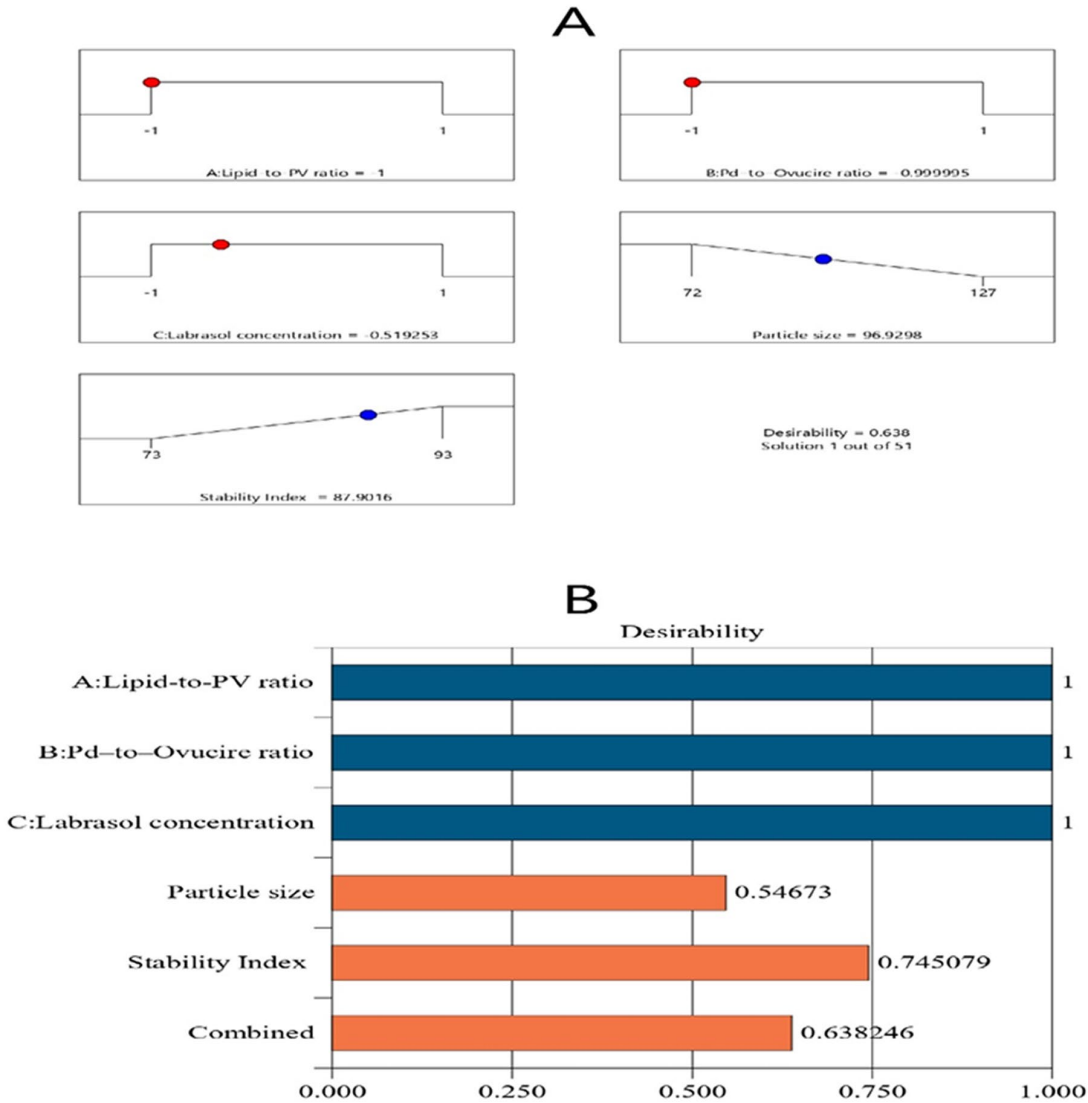
Bar chart and desirability ramp for the optimization process. The desirability ramp shows the levels of study factors and expected values for the dependent variables of the optimized PV-Pd-NLCs (A). The bar chart shows the values of desirability for the conjugated responses (B).

The major objective of optimization is to find the most appropriate levels of the studied factors to get a formulation with the best characteristics (Leroux et al., 1995). The Design-Expert software employed the desirability function (*D*) to optimize the assembled data. The software proposed varying solutions with several combinations of the independent variables. The desirability plot offered a desirability value of 0.638. The suggested optimum formulation had a 5:1 ratio of lipid:PV and a 1:9 ratio of Pd:Ovucire®, in addition to 1.5% of Labrasol®. The formulation had a particle size of 98 ± 2.5 nm and a stability index of 89 ± 1.1%. Figure 4 shows the bar chart and desirability ramp that reveal the desirability values of the responses and the most favorable levels of independent variables and predicted values of the responses. As per Table 4, the divergences between the actual and expected values of the responses were tenuous (*p* > .05).

**Table 4.** Tentative and expected values of the optimized PV-Pd-NLCs.

Solution	Lipid: PV ratio	Pd: Ovucire® ratio	Labrasol® %	PS (nm)	Stability index (%)	desirability
Expected value	5:1	1:9	1.5	96.9	87.8	0.6380
Tentative value	5:1	1:9	1.5	98	89	0.6380

**Table 5.** Rheological parameters of plain Carbopol hydrogel and Carbopol hydrogel containing optimized PV-Pd-NLCs.

Formulation	Viscosity^a^ (maximum) (cP)	Viscosity^a^ (minimum) (cP)	Farrow’s constant (N)	Area of hysteresis loop (Dyne/cm^2^.sec)	Flow behavior
Plain Carbopol hydrogel (F)	161,258 ± 25,612	10,456 ± 1,324	2.7506	132,232.80	Pseudoplastic
Carbopol hydrogel containing optimized PV-Pd-NLCs (A)	188,543 ± 34,754	12,544 ± 2,099	1.8202	167,606.10	Pseudoplastic

^a^ Data are expressed as mean ± SD (*n* = 3).

### 3.4. Ultra-morphological analysis of optimal PV-Pd-NLCs

**Figure 5.**
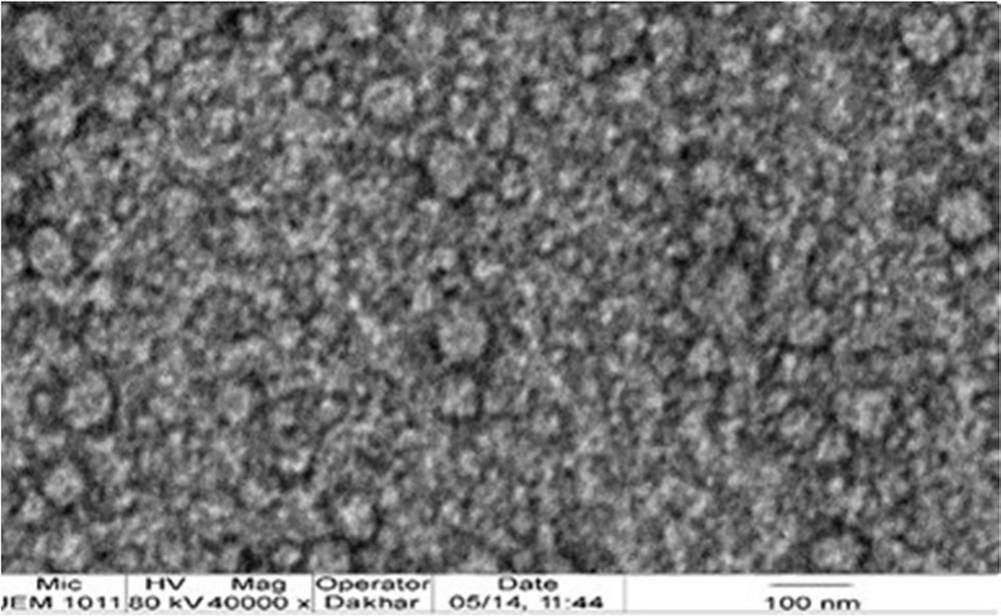
TEM photomicrograph of the optimal PV-Pd-NLC.

Figure 5 shows the TEM photomicrograph of the optimal PV-Pd-NLCs. The TEM image reveals that the nanocarriers have a spherical morphology with a well-defined outer membrane and that the particle size in the image is somewhat consistent with the particle size shown in the analysis. Moreover, the TEM image shows the homogenous distribution of the optimal formulation.

### 3.5. Determination of EE% and *in vitro* dissolution efficiency

The optimal formulation entrapped the PV with an efficiency of 91 ± 2%; this clarified the high capability of the NLCs to contain the drug with high percentages and affirmed earlier theories that NLCs are good at carrying and delivering drugs of a hydrophilic or lipophilic nature. As previously reported, a liquid lipid becomes distributed quite well within an NLC’s shell (Hu et al., 2005). The shell, which was rich in liquid lipid, was very flexible and allowed for significantly greater solubility for lipophilic drugs. Therefore, the PV was easily entrapped in large amounts (MÜhlen et al., 1996).

### 3.6. *In vitro* release study

**Figure 6.**
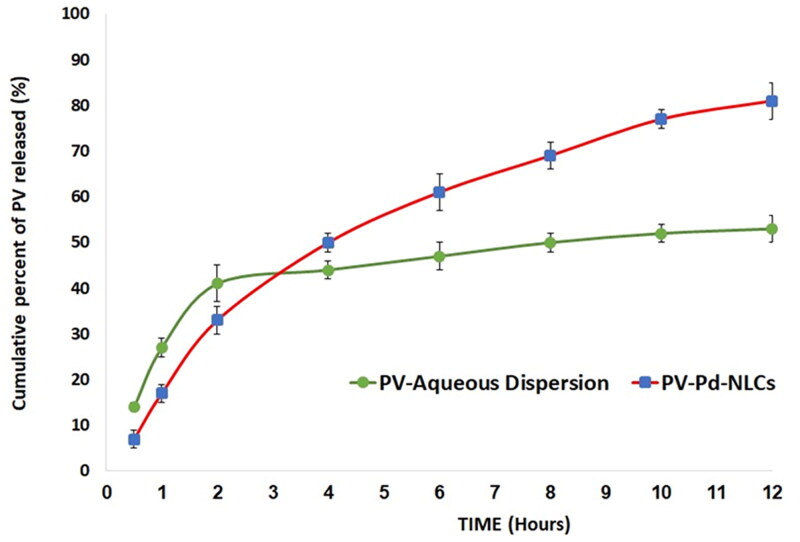
In vitro release profiles of PV from optimal PV-Pd-NLCs and PV suspension (mean ± SD, *n* = 3).

As for the *in vitro* dissolution behavior of the optimal formulation, it was found that it released 88 ± 3% of the loaded PV after 12 h; the drug suspension released only 53 ± 3% of the PV within the same time period. The enhanced release of the NLCs could be due to its liquid lipid content, which might contribute to its flexibility and enhance the drug diffusion and release into the medium (Jenning & Gohla, 2001). Other investigators suggested that loading a drug into an NLCs might transform the drug into its amorphous form and, hence, boost its dissolution and release compared with the crystalline drug suspension (Elmowafy et al., 2017). Furthermore, the surfactant content of NLCs might also encourage drug solubility and release into the surrounding medium via decreasing the interfacial tension between the particles and the surrounding medium; similar outcomes were reported (Elmowafy & Al-Sanea, 2021). Figure 6 presents the *in vitro* release profile of the PV from the optimal formulation and a plain drug suspension.

### 3.7. Characterization of the optimized PV-Pd-NLCs gel

#### 3.7.1. Rheological evaluation of the optimized PV-Pd-NLCs gel

**Figure 7.**
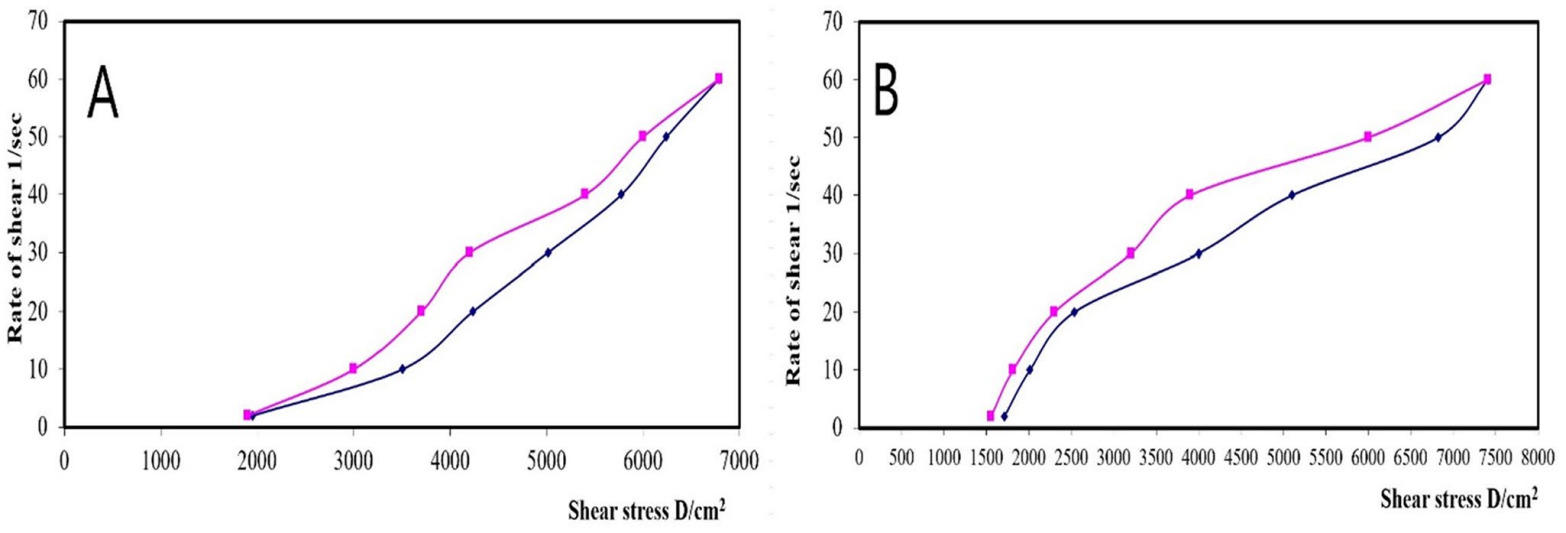
Plots of log shear rate (*G*) versus log shearing stress (*F*) for determination of Farrow’s constant (*n*) of (A) plain gel and (B) gel containing optimal PV-Pd-NLCs.

**Figure 8.**
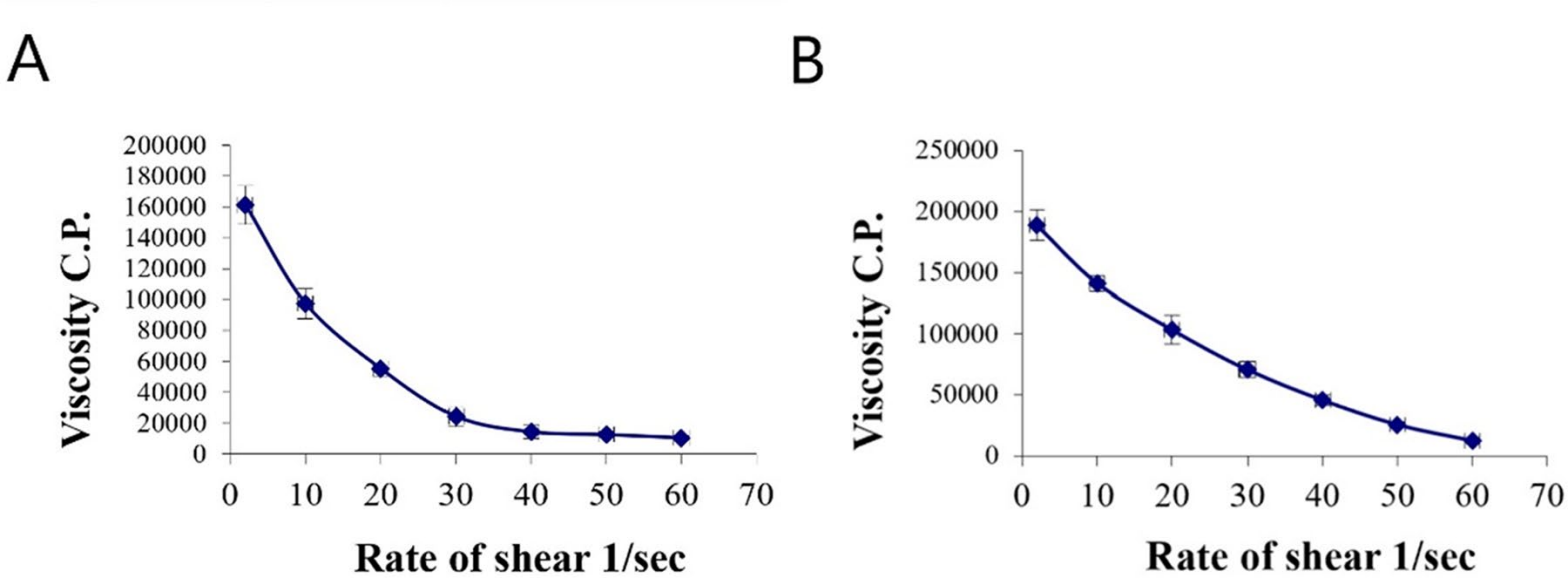
Flow curves of (A) plain gel and (B) gel containing optimal PV-Pd-NLCs.

**Figure 9.**
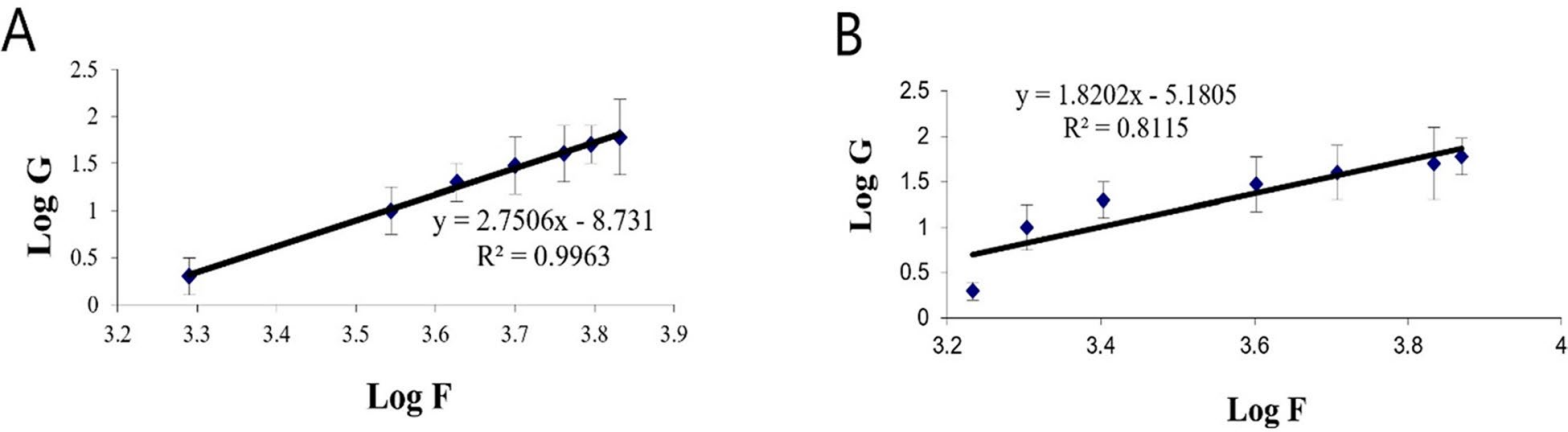
Rheograms of (A) plain gel and (B) gel containing optimal PV-Pd-NLCs. N.B.: The tested formulations were (A) hydrogel containing optimized PV-Pd-NLCs, (B) hydrogel containing NLCs formulated with castor oil instead of Pd oil, (C) hydrogel containing Pd-NLCs formulated without PV, (D) aqueous dispersion PV-Pd-NLCs formulated without Carbopol 940 gelling agent, and (E) Physical mixture of PV and Pd oil.

The rheological property of a semisolid drug carrier is a very important physical parameter for its application. As stated in Table 5, the optimal PV-Pd-NLCs-containing gel exhibited higher viscosity than the plain gel; this might be due to the presence of the nanocarrier. Plots of the log shear rate (*G*) versus the log shearing stress (*F*) were constructed for the determination of Farrow’s constant (*n*) (Figure 7), and it was found that both gels exhibited a pseudoplastic flow with an *n* value greater than 1. As illustrated by the flow curves in Figure 8, the formulations had good thixotropy and their viscosity decreased with an increasing shear rate. Thixotropy is the lessening of viscosity when an increased shear stress is applied to a substance such as a gel; when the shear stress is removed, the viscosity slowly returns to its original state under isothermal conditions (Lippacher et al., 2002). Complete rheograms were constructed by plotting the shearing rates as a function of the shear stresses (Figure 9). The figure shows a counterclockwise hysteresis curve, called a hysteresis loop. The gels had a pseudoplastic flow with thixotropic behavior. The degree of thixotropy was calculated quantitatively by determining the area of the hysteresis loop formed between the up and down curves of the rheograms using the trapezoidal rule. The gel containing optimal nanoformulation had a greater area of hysteresis loop, suggesting that it had superior thixotropic behavior.

#### 3.7.2. *In vitro* release of PV from different hydrogel formulations

**Figure 10.**
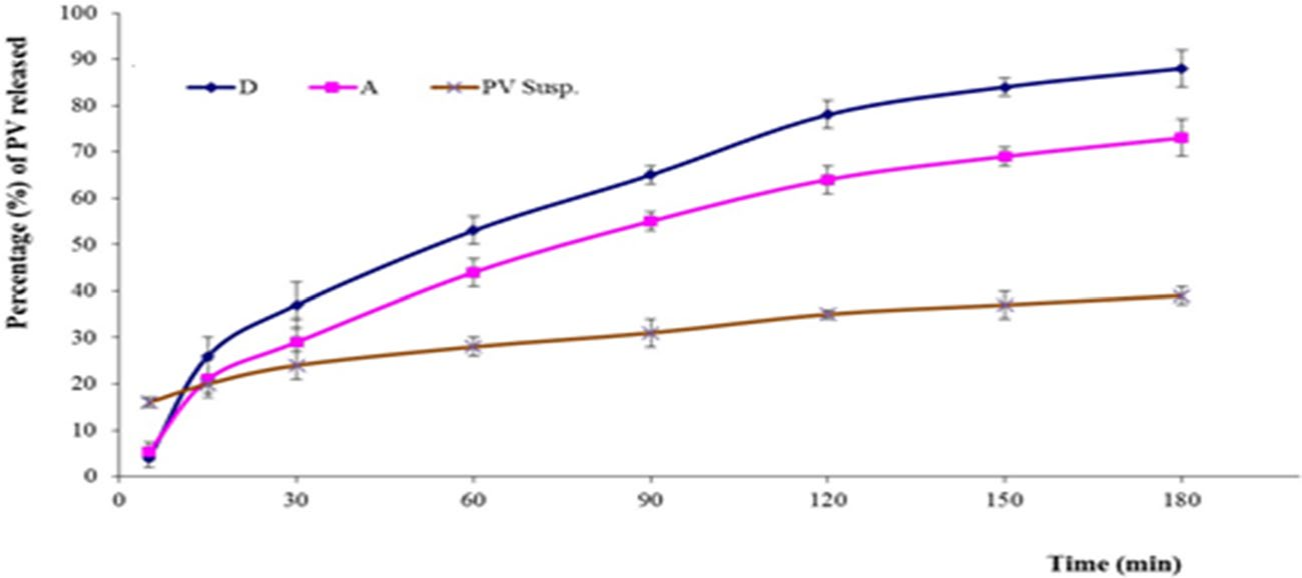
*In vitro* release profiles of PV from (A) gel containing optimal formulation, (D) aqueous dispersion of optimal formulation and drug aqueous dispersion (means ± SD).

As per the *in vitro* release study results, the hydrogel containing optimized PV-Pd-NLC (i.e. formulation A) released 73 ± 4% of the loaded PV, while the aqueous dispersion of the PV-Pd-NLCs (i.e. formulation D) released approximately 88 ± 3% of the loaded PV dose. The aqueous dispersion of the PV (1% wt/vol) released only 39 ± 2% of PV. The PV release behavior of the studied formulations is shown in Figure 10. As previously mentioned, the improved PV release from the aqueous dispersion of optimal NLCs could be due to its liquid lipid content, which might have contributed to its flexibility and enhanced the drug’s diffusion into the release medium, in addition to the suggested transformation of the PV into its amorphous form, which might have improved its dissolution. Moreover, the surfactant content of the NLCs might have also boosted the PV’s solubility and release into the surrounding medium via decreasing the interfacial tension between the particles and the surrounding medium (Jenning & Gohla, 2001; Elmowafy & Al-Sanea, 2021).

The observed decrease in drug release from the gel containing optimal formulation compared with the aqueous dispersion of the same formulation could be ascribed to the increased viscosity of the formulation due to the polymers used; this might have somewhat hindered the drug release (Rizg et al., 2022).

### 3.8. *In vitro* cell viability assay

**Figure 11.**
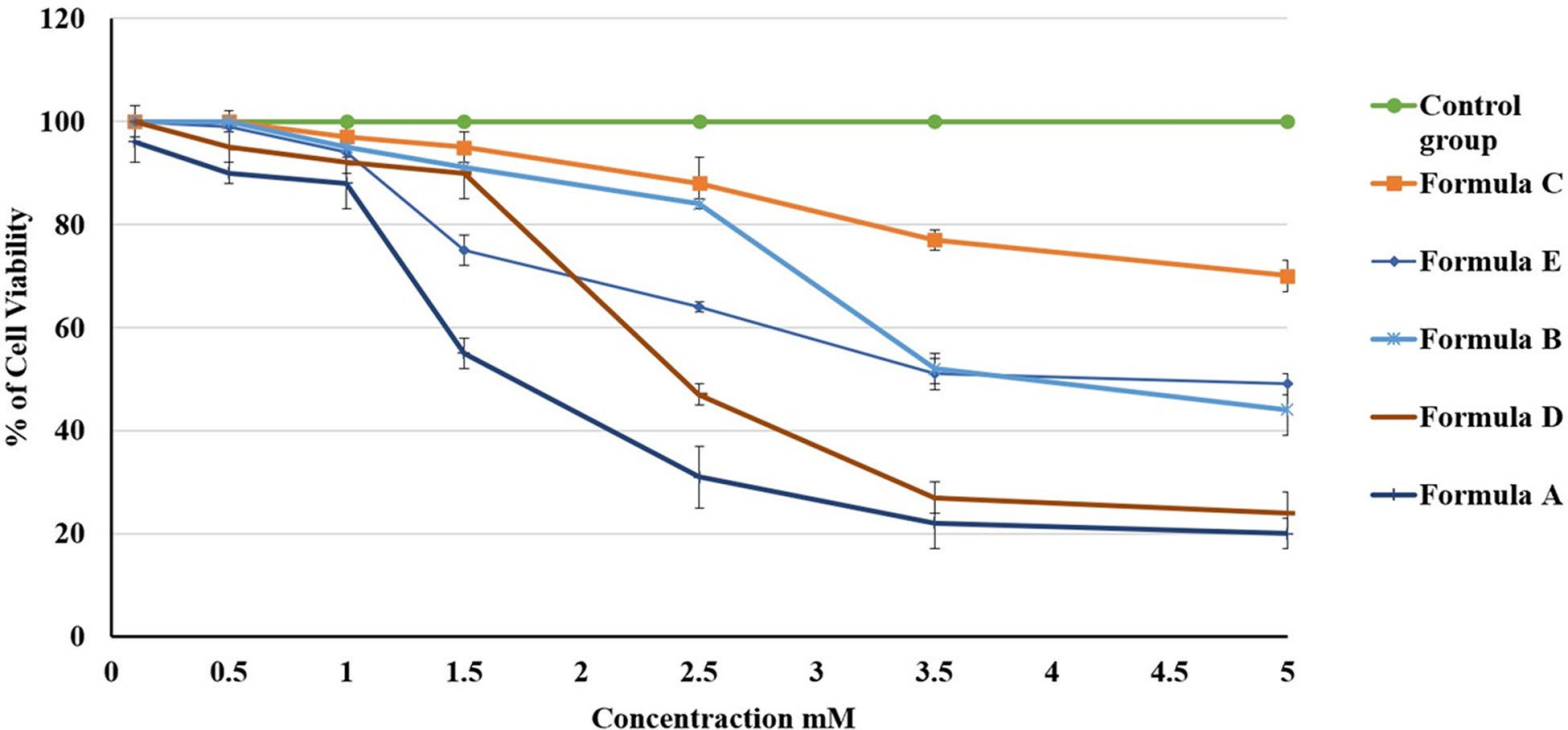
Effect of different PV-containing formulations and the control on the viability of the HGF-1 cell line. The values are the mean ± SD of three independent experiments.

The MTT test was utilized for estimating the *in vitro* cytotoxic behavior of formulations A, B, C, D, and E (see Table 2) against the gingival carcinoma HGF-1 cell line after 24 h of incubation. Figure 11 shows the variations in cell viability between the tested formulations; the cell viability was considered to be 100% for the negative control group. Figure 11 affirms that all of the formulations had decreased cell viability in a dose-dependent manner. IC_50_ values were 100%, 70 ± 3%, 49 ± 3%, 44 ± 5%, 24 ± 4%, and 20 ± 2% for the control group and formulations C, E, B, D, and A, respectively.

The PV-Pd-NLCs nanogel (formulation A) had a very low value of IC_50_ in comparison with formulations B, C, and E. This outcome signified the outstanding antiproliferative activity of formulation A against HGF-1 cells, which could be explained by the high affinity of PV for the P-glycoprotein transporter and the expected high level of cell permeation (Bogman et al., 2001). As for formulation D (i.e. dispersion of optimal PV-Pd-NLCs), it attained an IC_50_ value that was somewhat higher than that of formulation A. This might be ascribed to the viscous merit of formulation A, which was due to the presence of the gelling agent; it might have allowed more intimate contact of the nanoformulation with the targeted cells and, hence, improved the PV penetration of the cells and increased the cytotoxic effect of the PV (Rizg et al., 2022). However, the variations in the IC_50_ values of formulations A and D were found to be insignificant (*p* > .05).

Interestingly, both formulation B, which contained castor oil instead of Pd oil, and formulation C, which contained no PV, had low IC_50_ values. Nevertheless, the values were higher than those of formulation A (PV-Pd-NLCs nanogel) and formulation D (PV-Pd-NLCs dispersion). These findings affirmed the synergistic antiproliferative activity of PV when it was combined with Pd oil. Removing either the PV or the Pd oil from the formulation greatly decreased the cytotoxic effect.

### 3.9. LDH enzyme release measurement

Determining LDH level is a well-established test for assessing cell viability. It allows for rapid, strong, reproducible conjectures about the suggested toxicity of compounds and drug candidates (Kaja et al., 2017). Basically, LDH activity is usually evaluated using a coupled enzymatic reaction in which LDH oxidizes lactate into pyruvate, which then reacts with iodonitrotetrazolium chloride (INT) to make formazan. Formazan is a water-soluble dye that can be easily detected by the colorimetric method of analysis, with an absorbance at 492 nm. Following cellular damage due to intercellular or intracellular events, LDH is routinely released from the cytoplasm into the extracellular environment. Its stability in cell growth media makes it an adequate indicator of the existence of cellular damage and toxicity (Decker & Lohmann-Matthes, 1988).

**Figure 12.**
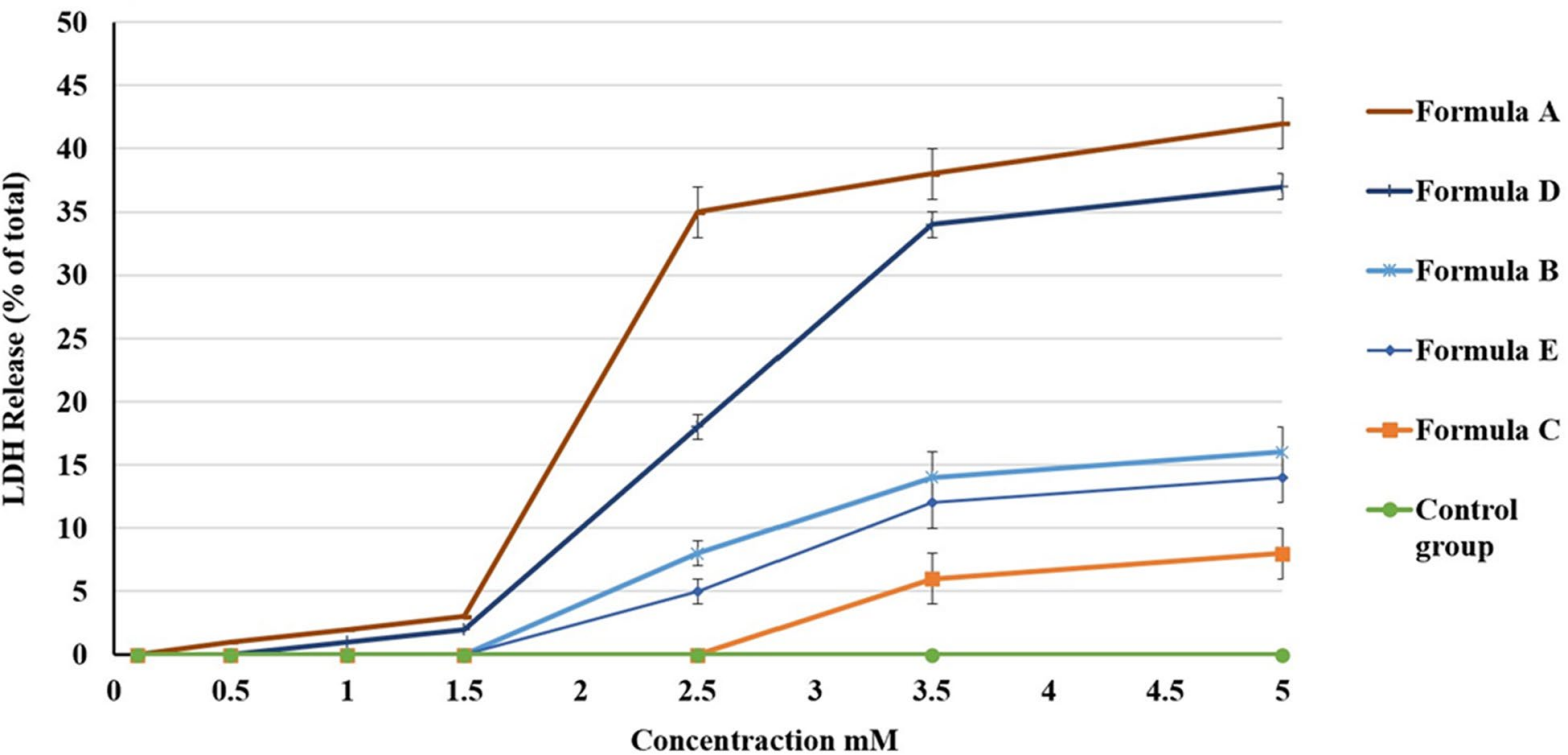
Effect of different PV formulations and the control on the percentage of LDH release from the HGF-1 cell line. The values represent the means ± SD of three independent experiments. N.B.: The tested formulations were (A) hydrogel containing optimized PV-Pd-NLCs, (B) hydrogel containing NLCs formulated with castor oil instead of Pd oil, (C) hydrogel containing Pd-NLCs formulated without PV, (D) aqueous dispersion PV-Pd-NLCs formulated without Carbopol 940 gelling agent, and (E) physical mixture of PV and Pd oil.

Figure 12 confirms that all of the formulations increased the release of LDH in a dose-dependent manner. Percentages of total LDH release were 42 ± 3%, 37 ± 2%, 16 ± 2%, 14 ± 2%, and 8 ± 1% for the A, D, B, E, and C formulations, respectively.

The high LDH ratio in formulation A could be due to the formulation’s viscous nature, which could have allowed more intimate contact with the cells. Formulation D had the second highest percentage of LDH released; this could be due to its content of the optimal formulation with no gelling agent. However, the difference between the abilities of formulations A and D to enhance the release of LDH was found to be insignificant (*p* > .05). Formulation B, which was composed of the optimal formulation but with castor oil instead of Pd oil, had a lower percentage of LDH release compared with formulations A and D. This signified the important synergistic cytotoxic effect of PV and Pd oil. Comparable results were reported in the literature (Ibrahim et al., 2019).

Formulation B had a higher percentage of LDH release than formulation E, which was composed of a mixture of PV and Pd oil. This outcome clarified the superior ability of the nanocarrier to deliver the candidate drug to the cells. This might be due to its surfactant content, which might decrease the interfacial tension between the carrier and the cells, and also to its liquid lipid content, which increases the membrane’s fluidity and enhances its cell permeation (Rizg et al., 2022). Formulation C, which contained a hydrogel loaded with Pd-NLCs formulated without PV, had the lowest percentage of LDH release; this finding could clarify the importance of PV as a cytotoxic agent and show that the main cytotoxic effect of the nanoformulation was due to PV. Figure 12 illustrates the percentage of LDH release of the different formulations.

## 4. Conclusion

PV was infallibly formulated as an NLCs with appropriate features. The most suitable lipid for formulating NLC was found to be Ovucire®, based on a solubility study of PV in different lipids. The particle size of the NLCs ranged between 72 ± 1.5 and 120 ± 5.1 with reasonable homogeneity. The stability index of the formulations was 73 ± 1.7 to 91 ± 2.0%. The optimized formulation had the proper stability (89%) and a suitable particle size (98 nm). Moreover, the optimized PV-Pd-NLC formulation was effectively dispersed in a Carbopol 940® gel base to form a nanogel that had satisfactory pseudoplastic flow with thixotropic behavior. The nanogel loaded with the optimal formulation had enhanced antiproliferative activity against the HGF-1 cell line compared with the other formulations, as shown by its very low IC_50_ value and high percentage of LDH release. Thus, this study showed the synergistic cytotoxic effect of PV and Pd oil on gingival cancer cells and the great potential of such a combination in treating oral cavity tumors.
